# Mesenchymal Stem Cells in Burn Wound Management

**DOI:** 10.3390/ijms232315339

**Published:** 2022-12-05

**Authors:** Agnieszka Surowiecka, Anna Chrapusta, Maria Klimeczek-Chrapusta, Tomasz Korzeniowski, Justyna Drukała, Jerzy Strużyna

**Affiliations:** 1 East Center of Burns Treatment and Reconstructive Surgery, Medical University of Lublin, 21-010 Leczna, Poland; 2 Malopolska Burn and Plastic Surgery Center, Ludwik Rydygier Memorial Hospital in Krakow, 31-826 Cracow, Poland; 3 Chair and Department of Didactics and Medical Simulation, Medical University of Lublin, 20-093 Lublin, Poland; 4 Department of Cell Biology, Faculty of Biochemistry, Biophysics and Biotechnology, Jagiellonian University in Krakow, 31-826 Cracow, Poland; 5 Department of Plastic Surgery, Reconstructive Surgery and Burn Treatment, Medical University of Lublin, 20-059 Lublin, Poland

**Keywords:** stem cells, burn wound, adipose-derived stem cells, burns

## Abstract

Mesenchymal stem cells have a known regenerative potential and are used in many indications. They secrete many growth factors, including for fibroblasts (FGF), endothelium (VEGF), as well as 14 anti-inflammatory cytokines, and they stimulate tissue regeneration, promoting the secretion of proteins and glycosaminoglycans of extracellular matrices, such as collagen I, II, III, and V, elastin, and also metalloproteinases. They secrete exosomes that contain proteins, nucleic acids, lipids, and enzymes. In addition, they show the activity of inactivating free radicals. The aim of this study was an attempt to collect the existing literature on the use of stem cells in the treatment of a burn wound. There were 81 studies included in the analysis. The studies differed in terms of the design, burn wound model, source of stem cells, and methods of cellular therapy application. No major side effects were reported, and cellular therapy reduced the healing time of the burn wound. Few case reports on human models did not report any serious adverse events. However, due to the heterogeneity of the evidence, cellular therapy in burn wound treatment remains an experimental method.

## 1. Introduction

**Figure 1:**
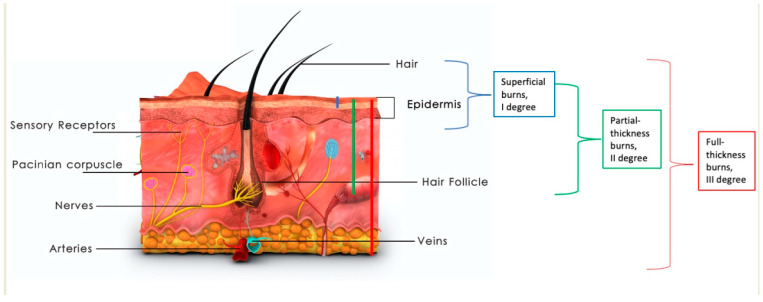
Depth of thermal burn. Images purchased under the Adobe Stock License.

Large burns, over 20% of total body surface area (TBSA), are a life-threatening condition due to loss of skin barrier and, in consequence, loss of fluids, severe metabolic changes, and infectious complications. In full-thickness burns, the entire depth of the skin is damaged, including the dermis, glands, and hair follicles. Only partial-thickness burns have the potential to heal spontaneously, as shown in Figure 1 [1]. Deep burn wounds have limited potential to self-heal, and in most cases require the removal of necrotic tissues followed by skin grafting or application of skin substitutes. In clinical practice, deep burns require removal of the burn eschar and replacing burned skin with skin grafts or skin substitutes. When large surfaces of skin are burned, one of the most commonly occurring problems is the lack of autologous skin to cover the burn wound. Many dressings and skin substitutes are available on the market; however, there is no one ideal solution. Regenerative medicine and stimulation of tissue repair is a growing discipline of medicine [2]. The amount of scientific evidence available for the usage of biostimulants, growth factors, and tissue-derived cells is increasing. Although it has been shown that EGF and HGF improve re-epithelialization, TGFb3 prevents scar contraction, and VEGF, PDGF, and FGF stimulate neovascularization, topical application of these growth factors, either separately or in combination, did not prove sufficient to improve burn wound healing. Even thought, it has been proved that epidermal growth factor (EGF) and hepatocyte growth factor (HGF) improve re-epithelialization, TGF-β3 prevents constricting scarring, whereas vascular endothelial growth factor (VEGF), platelet-derived growth factor (VEGF), and fibroblast growth factor stimulate neovascularization [3]. That is why it is cellular therapy and stem cells derived from different sources that might be the future of burn wound treatment and scar prevention [4].

Stem cells can be divided according to the ability to differentiate and mature. Embryonic and placenta are totipotent and pluripotent stem cells which are able to differentiate into cells of the three embryonic germ layers: endoderm, mesoderm and ectoderm. Mesenchymal stem cells are multipotent with reduced differentiation capacity into certain cell types. Unipotent stem cells give rise to only one type of cells, such as muscle or epithelial stem cells, and they participate in self-renewal processes and tissue regeneration. Mesenchymal stem cells (MSCs) are a type of undifferentiated cells with multipotent capacity that maintain and repair the tissue in which they are found and can differentiate into mature cell types such as adipocytes, osteoblasts, and chondroblasts [5]. Different names have been used for these cells over time, including “marrow stromal cells”, “multipotent stromal cells”, or “mesodermal stem cells” [6]. They are also referred to as “mesenchymal stromal cells” and described as fibroblast-like multipotent cells [7].

**Figure 2:**
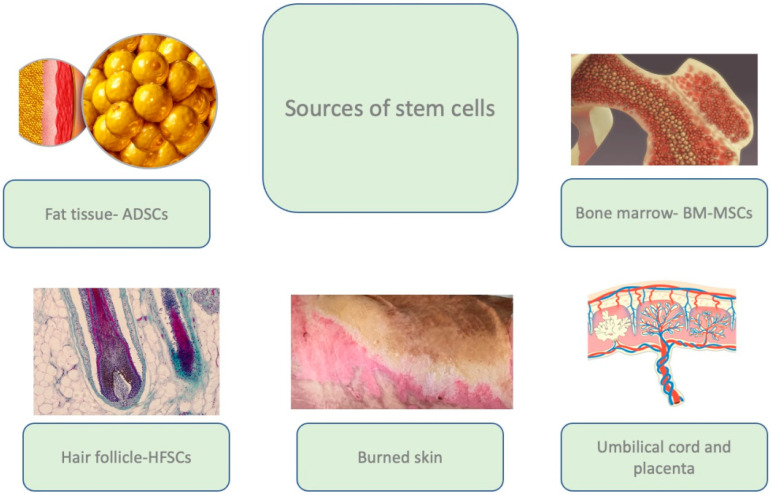
The main sources of adult stem cells used in bioengineering. Images purchased under the Adobe Stock License.

The regenerative potential of the MSCs, as well as accessibility, make cellular therapy a promising therapeutic option in wound treatment, including burn wounds. Adult stem cells are found in almost all adult tissues, including bone marrow, adipose tissue, the liver, the pancreas, the spleen, the thymus, skeletal muscles, dental pulp, and the dermis, with bone marrow and adipose tissue remaining the most frequently used sources of MSCs [5,7,8] (Figure 2).

## 2. Methods

Two independent researchers (AC, AS) screened articles available in medical databases (PubMed, PubMed Central, MEDLINE). The inclusion filters were “burn” or “burn wound” and “stem cells” or “MSCs” or “adipose-derived stem cells”. Titles, abstracts, and full texts in English were filtered to choose original articles and reviews describing the pathology of burn wounds and the role of residual stem cells, as well as the experimental and clinical use of stem cells in burn wound healing. The extensive heterogeneity of the methodology of the studies and the small number of comparable studies impeded reliable statistical analysis.

The search strategy identified 800 records, out of which 81 studies were included in the study. We performed a simple literature review.

### 2.1. Residual Stem Cells’ Reaction to Thermal Injury

**Figure 3:**
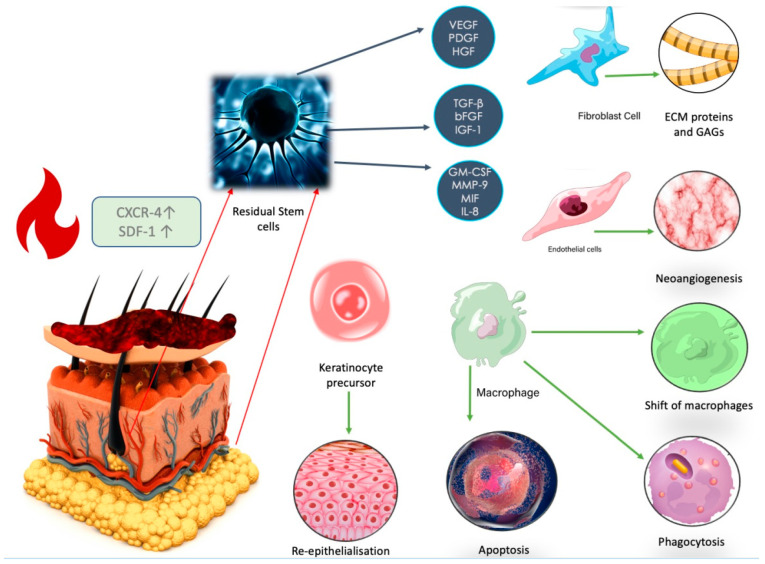
After trauma, expression of the CXCR-4 increases, and residual fat-derived stem cells from the area of the hair follicles, sweat glands, and nerve endings are recruited to regulate the local inflammatory response. Overexpression of the SDF-1 protein is responsible for cell migration in the early stages of wound healing. ADSCs promote the shift of the macrophage population from M1 to M2 and stimulate the secretion of anti-inflammatory TNF and IL-10. Images purchased under the Adobe Stock License.

There are residual mesenchymal stem cells (MSCs) in the dermis that are responsible for skin repair. They are present in niches in the region of the hair follicle bulges, at the insertion of pili muscles [9,10], sweat glands, nerve endings [11,12,13], and sebaceous glands [14]. With age, the structure of the skin changes and the number of stem cells decreases, which may lead to prolonged healing in elderly burn patients [15]. After burn trauma occurs, cytokines, such as tumor necrosis factor-alpha (TNF-α), interleukin (IL-1β), interferon IFN-γ IL-6, and IL-12 [16] are secreted. Stem cells can translocate to the injured epithelium and stimulate regeneration [10,17,18]. The process of cell translocation is promoted by increased expression of the CXCR-4 molecule on the MSC surface and stromal cell-derived factor 1 (SDF-1) protein [19]. The expression of SDF-1 is down-regulated by TGF-β1 and steroids [19]. In the initial phases of wound healing, MSCs promote a shift between the macrophage population from M1 to M2 and stimulate the secretion of anti-inflammatory TNF [18,20]. In the later phases, MSCs stimulate angiogenesis by secreting growth factors including vascular endothelial growth factor (VEGF), platelet-derived growth factor (PDGF), hepatocyte growth factor (HGF), fibroblast growth factor (b-FGF), SDF-1, transforming growth factor β (TGF-β), angiopoietin-1, and growth differentiation factor 11 (GDF11) [21]. MSCs have a crucial role in regulation of the inflammatory response via paracrine action through extracellular vesicles [22]. Exosomes contain signal-active substances, such as nucleic acids, amino acids, and proteins. Exosomes secreted by MSCs also contain mRNAs, such as miR-125a, which accelerate vessel formation [21]. In the proliferation phase, MSCs promote the secretion of ECM proteins, glycosaminoglycans, endogenous hyaluronic acid (HA), collagen I, II, III, and V, oxylatane, fibronectin, and elastin [23] by stimulating fibroblasts [20]. Secretion of growth factors is regulated by hosphatidylinositol 3 kinase/Protein kinase B (PI3K/AKT) [24,25]. Inhibition of PI3K/AKT/mTOR signaling promotes the process of autophagy [26]. MSCs. Effect on metalloproteinases (MMPs) and ECM remodeling is presented on Figure 3.

MSCs play an important antioxidant and antiapoptotic role [27,28]. Insulin-like growth factor 1 (IGF-1) inhibits apoptosis by the PI3K signaling pathway [21]. Interleukin-6 (IL-6) reduces oxidative stress by promoting transcription activator 3 (STAT3), nuclear factor 2 erythroid factor 2 (Nrf2), and superoxide dismutase (SOD). Nrf2 protects lipids from peroxidases by NOX1, whereas NOX4.MSC-secreted exosomes downregulate wound expression of NADPH oxidase isoform 1 (NOX1), NADPH oxidase isoform 4 (NOX4), interleukin 1β, interleukin 6, and tumor necrosis factor-alpha levels [21]. Glutathione peroxidase (GPx) and catalase inhibit the secretion of myeloperoxidase (MPO) [23,27,28], and stimulate BCl-2 expression, thus preventing hypoxia. Exosomes promote healing also by Wnt/β-catenin signaling [21]. MSCs inhibit NF-κB activation in TCR-stimulated T cells via the PDL1/PD-1 and Gal-9/TIM-3 pathways, playing an important immunomodulatory role in the wound healing process [21].

At the basal membrane, there is also an important population of residual keratinocyte stem cells, responsible for keratinocyte maturation [10]. They are characterized by the expression of α1, α2, α4, α6, β1, and β4 integrin, transferrin receptors, leucine-rich repeats and immunoglobulin-like domain proteins, and ATP-binding cassette subfamily G member 2 on their surface [29]. A decrease in integrin β1 in the stratum basale enables the migration of epidermal stem cells to the injury site [19,30]. After trauma, junctions between the basal layer of the epidermis and keratinocyte stem cells with integrin3β1 and laminin-5 play an important role [14].

### 2.2. Potential Use of Stem Cells in Burn Wound Healing

The majority of the studies on cell therapy in burns involve animal experiments (Table 1). According to a meta-analysis of 22 preclinical studies on 595 animals, stem cell therapy can significantly improve wound healing, especially in second-degree burns. Hair follicle-derived stem cells were the most efficient type of cells [31]. Similar findings were obtained by Yi et al. in an analysis of 20 studies with MSC therapy in burn wounds in animals, where stem cell treatment improved closure, reduced wound area, and improved vascularization of the tissues [32]. The most common form of stem cell application were intradermal injections in the wound edge or wound bed. However, there are also potential ways of cell application that do not involve injection: topical cells alone, cells encrusted in dressings, and topical treatment with an ointment, or intravascular treatment. The time from induction of the burn and wound debridement to administration of the cells varied significantly across the studies. In some of the studies, administration was performed immediately after the thermal injury, whereas in others, cell treatment was delayed for up to several days and preceded by burn wound excision. Most of the studies achieved a statistically significant improvement in wound healing in groups of animals treated with stem cells from various origins and with different forms of administration [33]. Abbas et al. compared the quality and effectiveness of autogenous stem cells obtained from bone marrow, adipose tissue, and dental pulp in a rat model. In the results, the most favorable myeloperoxidase activity of the stem cells was observed in the ADSC group [34]. Autologous ADSC accelerated wound healing and reduced the healing time, even to 15 days for deep burns [35].

Human studies are still limited and involve primarily case reports. Different types of stem cells were successfully used to reduce the burn wound area and improve healing time as well as prevent contracting scarring (Table 2).

No serious side effects were reported in the studies, which may suggest safety of the therapy. However, different types of stem cells (umbilical cord, bone-marrow, adipose-derived) were used and of different origins (autogenic, allogenic). Heterogeneity of the studies and case reports impede a decent comparison of the described therapies. Each stem cell type is characterized by individual features, but in vivo they act similarly as regulatory cells.

### 2.3. Umbilical Cord and Placental Stem Cells

Acquisition of umbilical-cord stem cells and placental stem cells is not painful and can be performed after placenta delivery [60,61]. Umbilical cord and placental stem cells can be used topically, in injections into the wound or intravascularly. Research studies reported usage of allogenic and xenogeneic stem cells for burn wound healing.

Human amniotic mesenchymal stem cells injected subcutaneously into thermally injured mouse skin reduced thermal stress-induced apoptosis by activation of PI3K/AKT/mTOR signaling and GSK3β/β-catenin. They also promoted the proliferation of dermal fibroblasts and keratinocytes, as well as the formation of tubular structures within the first 6 h post-burn [36].

Overexpressing IGF-1 human placental stem cells with IGF-1 injected into the burned mouse skin reduced the apoptosis rate and increased the proliferation rate. They also reduced the levels of pro-inflammatory cytokines, improved wound healing, and accelerated shrinkage of the burn area. Skin specimens from the injected wound contained fewer inflammatory cells and collagen fibers than the control specimen [37].

Human umbilical cord mesenchymal stem cells injected into the tail vein of rats influenced phosphorylation levels of P38MAPK and NF-B P65 proteins in the liver to inhibit the inflammatory response by reduction of secretion of HMGB, IL-6, and TNF-α. They also caused a shift to an anti-inflammatory M2 population of macrophages in the skin graft [38]. IL-6 and IL-1β increase blood–brain barrier permeability and induce neurological syndromes in severe burns. Umbilical cord-derived mesenchymal stem cells injected into the tail vein of mice reduced blood–brain barrier permeability and decreased levels of IL-6 and IL-1β 3 h post-burn [39].

A human study reported a case of amniotic stem cells being used. Kitala et al. presented the case of a 40-year-old female with an extent of deep burns (IIb°/III°/IV°) of 37% TBSA. Amniotic stem cells were applied under an acellular dermal matrix (ADM) substitute. The placenta was obtained during a caesarean section, with the written consent of the donor. The specimen was examined to exclude HIV 1 and 2, hepatitis B virus (HBsAg and anti-HBc), hepatitis C virus (anti-HCV), and syphilis. ADM from the human dermis was obtained by decellularization with a 0.05% trypsin solution. After debridement of the burn wound, the stem cells were transplanted and covered with ADM. The wounds healed completely within 12 days. No side effects were reported [55].

Umbilical cord and placental stem cells were reported to be effective in improving wound healing. The acquisition process, from healthy voluntary donors, may impede the widespread usage of the cells on larger number of patients.

### 2.4. Bone-Marrow-Derived Stem Cells

BM-MCSs injected in the rat tail vein were able to migrate to the burn wound and were observed in the wound bed up to day 7. Increase in neoangiogenesis, as well as CD31 and α-SMA was observed. After the systematic use of BM-MCSs reduction of levels of IL-1α, IL-2, IL-6, and interferon (INF)-γ can be detected [62].

BM-MCSs injected subcutaneously can accelerate secretion of the epidermal growth and granulation formation. Other effects may include downregulation of pro-inflammatory cytokines, downregulation of TGF-β, IL-6, TNF-α, MMP-9, and microRNA21 [40]. Injected allogenic BM-ADSC increased secretion of chemokine ligand 2 (CXCL2), granulocyte macrophage-colony-stimulating factor (GM-CSF), L-selectin, intracellular cell adhesion molecule (ICAM)-1, tissue inhibitor of metalloproteinase (TIMP)-1, and interleukin (IL)-4. Migration of the labeled BM-ADSCs was detected in the burn wound after 7 days. α-SMA and CD31 were elevated. Systemic levels of IL-1α, IL-2, IL-6, and interferon (INF)-γ levels were lower in the treatment group [41].

In a different model, in adult male Wistar rats, BM-MSCs combined with simvastatin improved wound healing. BD-MSCs were injected intradermally, and simvastatin was administrated intravascularly. In the study, the observed levels of α-SMA, CD31 and VEGF genes in granulation tissues were also significantly higher. In the qRT-PCR findings, the expression levels of Akt and mTOR transcripts were higher [42]. Overexpression of caveolin-1, the structural component of the caveolae membrane important in cell signaling, improved the efficacy of BD-MSCs and decreased serum levels of IL-1, IL-6, and TNF-α [43].

Rasulov et al. in 2005 described a case of a burn in which they applied stem cells. The wounds, of intermediate and full thickness, covered up to 40% of the body surface. Despite several surgical cleanses of the wounds and skin grafts, healing was not achieved. Rasulov used allogeneic stem cells obtained from two healthy volunteers, from the iliac plate. The cells were applied to the wound surface and after a few days the skin was re-transplanted, this time achieving complete wound closure [56].

There are reports of similar use of bone marrow stem cells collected from a deceased donor [57]. Lataillade successfully treated radiation burns using autogenous myeloid stem cells and a collagen matrix [58]. Mansilla et al. examined the concentration of stem cells in the peripheral blood of burned and healthy volunteers, observing a positive correlation between the number of cells and the extent of the burn. In younger patients, the number of stem cells was higher than in older patients [57].

Jeschke et al. reported a case of a patient with full-thickness burns covering 70% of the body surface, in whom allogeneic myeloid stem cells were used. After cleaning the burn wound, they applied stem cells and fibrin glue to the surface of the burn wound, and then disassembled the allografts. Approximately half of the grafts healed. In the next procedure, the edges of the wound were injected with a commercial suspension of allogeneic myeloid stem cells and the allografts were disrupted, resulting in the closure of approximately 90% of the wounds [59].

### 2.5. Adipose-Derived Stem Cells

The California Institute for Regenerative Medicine has defined adult stem cells as cells that “are committed to becoming a cell from their tissue of origin and can’t form other cell types” [63]. The definition of pluripotent stem cells describes cells having “the potential of taking on many forms in the body, including all from the more than 200 different cell types” [63].

However, many authors have repeatedly demonstrated that adipose-derived regenerative cells and adipose-derived stem cells can indeed form other cell types, owing to a three-germ layer differentiation capacity [63]. To standardize MSCs, in 2006 the International Society for Cell and Gene Therapy proposed the minimal obligatory criteria:

(1) Trilineage potential presenting in the ability to differentiate into adipocytes, chondrocytes, and osteocytes in vitro;
(2) Expression of the surface markers CD73, CD90, and CD105;
(3) Lack of expression of hematopoietic and endothelial antigens CD14 (or CD11b), CD19 (or CD74alfa), CD 34, CD 45, and HLA-DR surface markers;
(4) Plastic adherence in standard culture conditions [5,6,7].

The immunophenotype of AD-MSCs is more than 90 percent identical to bone-morrow mesenchymal stem cells (BMSCs), with the major difference in the presence of the glycoprotein CD34 on the AD-MSCs cell surface [5]. Except for the abovementioned surface markers, expression AD-MSCs are positive for pericyte markers CD140a and CD14d and the smooth muscle marker alfa-smooth muscle actin [5]. The presence of the pericyte marker suggests a possible niche for AD-MSCs within the perivascular region of fat tissue, near small capillaries, between mature adipocytes and the extracellular matrix [5]. Most often, a flow cytometer with specific stem cell antibody marker (fluorescent antibody cell sorting) is used to identify the cells, while gene expression is performed with quantitative real-time polymerase chain reaction (RT-PCR) and microarray analysis.

AD-MSCs have immunomodulatory and paracrine functions as well as the ability to secrete a wide range of bioactive molecules, such as cytokines, chemokines, and antioxidants [7,64]. They produce growth factors such as vascular endothelial growth factor, hepatocyte growth factor, fibroblast growth factor 2, and IGF-1, which are important in the initiation of angiogenesis, adipose tissue regeneration, and in overall healing processes [5,7,64,65].

The immunomodulation function of AD-MSCs consists of inhibition of dendric cell differentiation by prostaglandin E2, and suppression of immunoglobulin synthesis. Promotion activity is aimed at anti-inflammatory M2 macrophage polarization and regulatory T-cell proliferation. Secretion of chemokine (C-C motive), ligand 2, and indoleamine 2,3-dioxygenase inhibits the synthesis of immunoglobulin by B cells. The aforementioned T-cell proliferation is regulated by leukemia inhibitory factor, hepatocyte growth factor, interleukin 10, prostaglandin 2, tumor growth factor-beta, and indoleamine. Inhibition of CD8+ and CD4+ T lymphocytes and natural killer cell proliferation is regulated by indoleamine 2,3-dioxygenase, nitric oxide, interleukin 6, prostaglandin 2, and tumor growth factor-beta [7].

The great advantage of adipose tissue stem cells is their availability and the technical ease of obtaining them [35]. Although the first described source for harvesting stem cells was bone marrow, currently adipose tissue is assessed as the most precious resource in clinical practice. Apart from worse accessibility, bone marrow delivers cells with lower proliferative capacity, longer doubling time, and lower viability in comparison with adipose-derived cells. The frequency of AD-MSC is 500 times higher than that of BM-MSC, which is why it allows for the delivery of a large number of cells that can be obtained in many ways [66]. The type of the harvesting procedure applied influences the quality of AD-MSCs, which means the viability and ability for proliferation [66]. The most frequently used methods include aspiration, liposuction, and surgical excision [66,67]. Aspiration allows for easy and quick harvesting of a large amount of fat tissue while leaving a minimal scar. It can be performed as power-assisted vacuum aspiration or mechanical-assisted syringe aspiration, as described by Coleman [66]. The advantage of the Coleman technique is the reduction of trauma to the adipocytes owing to the gentle negative pressure applied in this method. A disadvantage of syringe aspiration is the longer duration of the procedure needed to obtain the same number of cells as in vacuum aspiration. Apart from the higher risk of cell trauma, a disadvantage of the vacuum method is that specialized equipment is required, according to the selected technique. Power-assisted liposuction (PAL), laser-assisted liposuction (LAL), and ultrasound-assisted liposuction (UAL) are the most commonly used approaches [66]. Surgical resection of fat tissue is a method of harvesting adipocytes without damaging them. This requires an incision of the skin, which leaves a post-surgical scar. Local anesthesia is sufficient for small procedures. If a large amount of adipose tissue is needed, the procedure should be performed under general anesthesia, which can be considered a disadvantage.

Autogenic, allogenic, and xenogeneic ADSCs are used in burn wound treatment. Topical use of allogenic ADSCs in a sheep burn model improved skin graft intake and blood flow in the burn wound, increased VEGF levels, and accelerated wound epithelialization after the excision of a full-thickness burn model [44]. Topical administration of allogenic ADSCs can reduce the extent of the wound and accelerate healing [45]. For topical use, ADSCs were often combined with dressings. Allogenic ADSCs can be incorporated in a cellulose membrane to treat second-degree burns [46] and can also be incorporated in PEG hydrogel [68] or 5% collagen hydrogel [47]. Hydrogel and ADSCs in combination were used in full-thickness thermal burns in a rat model and improved wound closure (95% vs. 79% with a saline gauze). They also stimulated granulation and remodeling of the dermal layer. Dong et al. treated deep second-degree burns in mice with a hydrogel dressing with hyaluronic acid with embedded human ADSCs. Significant improvement in healing in the treated group was observed on day 3. Examination of the specimens showed a higher number of vessels, a higher ratio of collagen type III to I, and a reduction in active myofibroblasts [48]. ADSCs can also be incorporated in 3D-printed scaffolds [49]. There are also reports of ADSC being suspended together with keratinocytes and fibroblasts on bioabsorbable nanostructures that could serve as composite grafts [69].

In some reports, ADSCs were injected into the burn wound or burn wound edges. In a study by Rezai et al., allogenic ADSCs were injected intradermally to improve wound healing and reduce the number of inflammatory cells in the wound. ADSCs promoted VEGF gene expression and secretion of TGF-β. ADSC also promoted the proliferation of dermal fibroblasts [50]. Injected intradermally, allogenic ADSCs reduced the area of the burn after 14 days as well as reduced lymphatic vessels and influenced the concentration of collagen type III [51].

Curcumin-preconditioned ADSCs had an increased capacity for migration, proliferation, and paracrine potential, suppressed secretion of pro-inflammatory cytokines, and improved healing in comparison with innate ADSCs [52]. In a rat burn model, autologous ADSCs were seeded in a PRP matrix and injected under the meshed skin graft to improve healing and neovascularization [70].

Intravascular injections of ADSCs might have an influence on organ burn-related changes. ADSCs injected intravascularly in a swine burn model of a 40% deep burn influenced prothrombin times and INR, wherein low doses revealed slight hypercoagulation. ADSCs reduced the partial thromboplastin time, fibrinogen, and d-dimer levels, which had been increased by the burn. Low doses of IV-ADSCs slightly increased creatinine [71].

ADSCs are easy to collect even from voluntary donors after elective liposuction. Banking ADSCs might be a promising option for cellular burn therapy in future.

### 2.6. Hair Follicle Stem Cells, Keratinocyte Progenitors, and Stem Cells from Excised Burned Skin

Allogenic hair follicle stem cells (HFSCs) obtained from rats and injected into deep partial-thickness burns accelerated wound healing and improved epidermal thickness. HFSCs stimulated neovascularization and increased the expression of proangiogenic CD31 on endothelial cells. The acquisition of HFSCs was performed by collecting a whisker from the upper lip and processing it with collagenase I/Dispase II solution. The bulges were then removed from the capsule and small pieces were cultured. Flow cytometric analysis was performed to determine the expression of CD34 and Nestin [53].

Human-induced pluripotent stem cells, derived from reprogrammed human skin fibroblasts, express CD200, integrin α-6 (ITGA6), integrin β-1 (ITGB1), transcription factor P63, keratin 15 (KRT15), and keratin 19 (KRT19)-like keratinocyte progenitor cells [10]. They can improve wound healing [72]

Xenogenic HFSCs were integrated into human acellular amniotic membrane to treat third-degree burns in mice. The composite did not change the volume of the stem cells and improves wound healing after day 7. Both HFSCs in a composite as well all HFSCs alone improved neovascularization in full-thickness skin defects [73].

Human MSCs obtained from excised burned skin with a third-degree burn injected in the burn wound improved healing in a mouse and swine burn model [54].

Dolp et al. obtained stem cells from discarded burned skin without subcutaneous fat. In comparison with umbilical cord stem cells, there was no significant difference in the expression of MHC I and II, ROS release, or colony formation [74]. Van der Veen also obtained stem cells from the eschar [75].

One of the threats in acquiring autologous stem cell from the eschar is the influence of the thermal destruction of all type of cells in deep burns. Additionally, in large burns over 20% TBSA, lesions due to the burn shock can be observed in many organs and the influence of the burn shock on stem cells is still unknown.

### 2.7. Stem Cells in Scar Prevention and Treatment

A scar is not only a cosmetic problem but most importantly a functional one. There are four stages of scar formation: hemostasis, inflammation (day 1–3), proliferation (day 4–21), and remodeling (from day 21 to a year) [76]. In the first stage, platelets, erythrocytes, leukocytes, and fibrin fibers are involved in the formation of a clot [77]. Then, neutrophils “flow” to the damaged tissue, as they are responsible for combating potential pathogens, debridement of the wound by phagocytosis, and the secretion of pro-inflammatory mediators and chemokines [76,77]. When they undergo apoptosis, monocytes migrate to the wound area, where they mature into macrophages [77]. Macrophages are important cells of the proliferation period [76]. They have regulatory properties and stimulate fibroblasts to secrete extracellular matrix proteins, including type III collagen. The most important cytokines in this period are PDGF and TGFβ 1 [76]. Moreover, macrophages stimulate the maturation of fibroblasts into myofibroblasts and secrete metalloproteinases. M1 macrophages are primarily pro-inflammatory, and an increase in their numbers in scar tissue is detected in the early phase, between 7 and 14 days. Re-epithelialization occurs after the stimulation and migration of keratinocytes [76]. In the scar remodeling phase, granulation tissue is formed, in which type III collagen is degraded and replaced with type I collagen [76]. M2 macrophages, with a suppressor effect, are detected in the late phases of scar remodeling (after 28 days). The activity of M2 macrophages, which persists for too long, is observed in hypertrophic scars. Reducing the number of macrophages and inhibiting their activity, prevented scar from overgrowth [78]. The pro-inflammatory cytokines IL-6 and IL-8 stimulate scar overgrowth, while anti-inflammatory cytokines, such as IL-10, have the opposite effect [76].

MSCs can be used to prevent the formation of contracting scars (Table 3). Piejko et al. used autogenic ADSCs in the combined treatment of post-burn contracting scar of the neck. In the experimental part of the study, a comparison of different types of cells seeded in the dermal substitute was performed. ADSCs were characterized by higher secretion of VEGF and pro-angiogenic interleukin 6 (IL-6) than human fibroblasts. The transcription of metalloproteinases 2 and 9 was also increased, which potentially can favorably influence the scar remodeling process and improve the quality of the scar. In the further reported case, a contracting post-burn scar on the neck was qualified for excision. Autogenic ADSCs were harvested by means of surgical excision of adipose tissue 3 weeks before the subsequent stage. Cells were isolated, cultured, and seeded into a dermis-substitute matrix based on bovine collagen and sulphite-chondroitin. Four weeks after scar excision, the silicone layer of the matrix was removed and the neodermis was covered with a split-thickness skin graft. No adverse events were reported. The skin in the area treated with ADSC-s-matrix was more elastic and less reddish than in non-treated areas [79].

Zahorec et al. described a case of post-burn hypertrophic scars corrected with surgical excision followed by lipofilling and injections of autologous isolated and cultured ADSCs. The obtained improvement in scar quality showed a reduction in Vancouver Scar Score (VSS) from 7.63 points to 2.38 at the 6-month follow-up [80].

**Table 3:** Clinical studies considering usage of autologous agents in post-burn scars.

Study	Study Type	Patients and Methods	Outcomes	Conclusion
Piejko [79]	Clinical experiment, case study	Contracting post-burn scar on the neck was qualified for excision. Autogenic AD-SCs were harvested by surgical excision of adipose tissue 3 weeks before the next stage. The cells were isolated, cultured, and seeded into the dermis substitute-matrix based on bovine collagen and sulphite-chondroitin. Four weeks after the scar excision the silicone layer of the matrix was removed and the neodermis was covered with a split-thickness skin graft.	No adverse events reported, good scar quality and texture.	Autologous stem cells can promote “scarless” healing of deep tissue wounds.
Li [81]	Experiment	Adipose and scar tissue was collected from subjects that underwent plastic excision of scars. Adipose-derived stem cells were isolated and cultured. Then, exosomes were isolated. BABL/c mice were randomly divided into groups; 3 days after creating a full-thickness injury, exosomes were injected subcutaneously.	Exosomes inhibited the proliferation and migration of fibroblasts, decreased the expression of Col1, Col3, α-SMA, IL-17RA, and p-Smad2/p-Smad3 and increased the levels of SIP1 in HSFs. miR-192-5p was highly expressed in ADSC-Exo and targeted the expression of IL-17RA to decrease the pro-fibrotic protein levels.	ADSC-Exo have antifibrotic features and improve wound healing.
Zahorec [80]	Clinical experiment	8 patients with post-burn scars: 2 keloid, 6 hypertrophic. Adipose tissue was harvested with the Coleman technique, then ADSCs were isolated and cultured. ADSCs were injected with a 20G needle, subdermally after scar resection.	Improvement in VSS score in a 6-month observation (7.63 to 2.38), elapsing from scar incidence to correction was 79 months	Autologous ADSCs are safe and effective in preventing and treating post-burn scars.
Meng [82]	Experimental	Fibroblasts from hypertrophic scars were co-cultured with umbilical cord stem cells.	Umbilical cord stem cells suppressed the proliferation and migration ability of fibroblasts, with the TGF β1/Smad3 pathway inhibited as well. Additionally, levels of mRNA of collagen type Iα2 (*COL1A2*), collagen III α1 (*COL3A1*) and actin α2 smooth muscle (*ACTA-2*) were lower.	Umbilical cord stem cells have anti-fibrotic potential.

Additionally, exosomes isolated from human adipose-tissue-derived stem cells can be used to improve scar quality. ADSC exosomes inhibited the proliferation and migration of fibroblasts and decreased collagen deposits. They also decreased pro-fibrotic markers, such as Col1, Col3, α-SMA, and IL-17RA. MiR-192-5p modulates gene expression to suppress IL-17RA [81]. Hypertrophic scar fibroblasts cultured with umbilical cord stem cells were characterized by a suppressed proliferation and migration ability. Furthermore, the TGF β1/Smad3 pathway was inhibited, and mRNA of collagen type Iα2 (*COL1A2*), collagen III α1 (*COL3A1*), and actin α2 smooth muscle (*ACTA-2*) were lower [82].

## 3. Conclusions

At this point, stem cell therapy in burns is at the experimental level. However, the first results are promising and understanding of stem cell biology is good, so more studies should be performed. Experimental studies on small and bigger animals showed that stem cells of different origins have a potential to accelerate burn wound healing. Just a few cases report on humans reported optimistic results and no side-effects. Due to limited evidence and randomized studies, the routine use of stem cells in the treatment of a burn wound cannot be recommended. Further studies on more patients should be performed to analyze the safety and efficiency of stem cells in burn wound management as well as to establish the most appropriate cell type, origin, and way of application. Research is needed to prove the viability of autogenous mesenchymal stem cells and their safety in use.

## Figures and Tables

**Table 1:** Experiment studies on animal models on the use of stem cells in burn wound healing.

Study	Study Type	Methods	Outcomes	Conclusion
Li YK [36]	Experiment, mice	Human amniotic mesenchymal stem cells were recruited, selected, and cultured from human fetal placentas obtained from the volunteers, and injected subcutaneously into thermally injured mouse skin.	A mean number of 2 × 10^6^ stem cells was injected. An increase in PCNA and CK19 was observed on days 7 and 14, with more tubular structures observed after the initial 6 h post-burn, as well as inhibited heat stress-induced apoptosis and promoted proliferation of dermal fibroblasts and keratinocytes and activated PI3K/AKT/mTOR signaling and GSK3β/β-catenin.	Human amniotic mesenchymal stem cells inhibit stress-induced apoptosis.
Cheng [37]	Experimental, mice	Human placental MCS were obtained. Using a lentivirus, overexpression of IGF-1 was obtained in MSCs; 96 mice were divided into 4 groups: control, burn, burn+unmodified MCS, burn + IGF-1modfied MCS. The cells were injected on days 1, 4, 8, 12, 16, and 20 after the burn injury with 2 × 10^6^ of hPMSC-Lv-Vector or hPMSC-Lv-IGF-1 at 4 points around the burn wounds.	Induction of epithelial differentiation was observed. Inhibition of cell apoptosis and stimulation of cell proliferation was observed in the IGF-1 group. Modified stem cells stimulated wound healing and in the skin specimen reduced inflammatory cells in the wound bed. They also reduced pro-inflammatory cytokine levels of IL-1b, IL-6 and TNF-a, as well as TGF-β1, collagen I and collagen III expressions in vivo, and increased VEGF levels.	Modified MSCs with overexpression of IGF-1 have the potential to promote faster wound healing and reduction of scar contraction.
Jian-Xing [38]	Experiment, rats	A 30% third-degree burn was created. After escharotomy, human umbilical cord stem cells were injected into the tail vein of rats.	Levels of IL-6 and TNF-α were lower, phosphorylation levels of P38MAPK and NF-B P65 proteins in the liver to reduce the inflammatory response, a shift to anti-inflammatory M2 population of macrophages in the skin graft.	Stem cells administrated intravascularly reduce inflammation by regulating liver secretion of proteins and cytokines. They improve skin graft healing and reduce scarring.
Yang [39]	Experimental, mice	Allogenic umbilical cord mesenchymal stem cells were injected into the tail vein in severely burned mice. The Dextran model was used to evaluate blood–brain barrier permeability.	UC-MSC reduced blood–brain barrier permeability and decreased levels of IL-6 and IL-1β in serum and in the brain.	Systematic injection of UC-MCS can improve the integrity of the blood-brain barrier and prevent neurological symptoms in severe burns.
Abdel-Gawad [40]	Experiment, rats	90 rats divided into three groups: control (6), burn model (42), and study (42). Bone-marrow stem cells were injected subcutaneously in the study group.	Decrease in wound contraction, downregulation of TGF-β, IL-6, TNF-α, MMP-9, and microRNA21.	Bone-marrow-derived stem cells improve healing of burn wound and reduce scar formation.
Li [41]	Experimental, rats	BM-MSC were harvested from rats and labeled with Fe_3_O_4_ NP. A full-thickness burn was created, and then stem cell were injected in the tail vein.	Labeled stem cells were non-toxic. Labeled stem cells migrated to the burn wound up to day 7. Increase in neoangiogenesis factors was observed: increase in CD31 and α-SMA. Reduction of systemic levels of IL-1α, IL-2, IL-6, and interferon (INF)-γ.	Intravascularly injected stem cells can migrate to the burn wound and improve healing. They reduce systemic levels of pro-inflammatory cytokines.
Ramhormozi [42]	Experimental, rats	Bone-marrow-derived stem cells (BMS) were obtained from adult male Wistar rats; 40 rats were burned and divided into groups: control, simvastatin iv, BMS intradermally, and BMS+ simvastatin. Wound healing, collagen, re-epithelialization were examined. Additionally, qRT-PCR for Akt/mTOR signaling pathway; CD31 and VEGF genes.	Better wound healing was observed in the group were stem cells were injected intradermally and simvastatin was administrated intravascularly. Additionally, levels of α-SMA, CD31 and VEGF genes in granulation tissues were also significantly higher. In the qRT-PCR findings, the expression levels of Akt and mTOR transcripts were higher.	A combined therapy improved healing by stimulating Akt/mTOR signaling pathway.
Wu [43]	Experiment, rats	Allogenic BM-CS were used. A model of a deep second-degree burn was created. A plasmid pLV-CMV-EF1-fLuc-T2A-puro was used to create a recombinant lentivirus with overexpression of caveolin-1 and transfected to BM-MSCs and injected intradermally 5 min after burn injury.	Overexpression of caveolin-1 improves the efficiency of BM-MCSs in burn wound healing and shortened the healing time to 10 days. The protein expression of TGF-b1, TGF-b3, FGF, and EGF was increased. Additionally, serum levels of IL-1b, IL-6 and TNF-a were decreased.	Application of exogenous MSCs overexpressing caveolin-1 improves burn wound healing.
Fujiwara [44]	Experimental, sheep	Allogenic ADSCs were obtained and administered topically; 7 sheep were enrolled into the study. After a burn and excision of a deep burn, the wound was covered with a skin graft (2 × 2 cm) and ADSCs were applied topically in the study group.	Topical use of allogenic ADSCs in sheep improved graft intake and wound blood flow, increased VEGF levels in the wound, and accelerated wound epithelialization after the excision of a full-thickness burn model.	Topical allogenic ADSCs accelerate graft intake and improve wound vascularization. A new burn model was established.
Hermeto [45]	Experiment, rats	Two rats were donors of adipose-derived stem cells. ADSCs were harvested, isolated, and cultures; 40 rats were divided into 4 groups: placebo gel, insulin gel, topical ADSCs, and topical ADSCs+ insulin gel applied on superficial second-degree burns.	ADSCs improved healing and reduced the wound extent in both topical ADSC and topical DSC+ insulin gel groups.	Topical use of ADSC can be useful in the treatment of superficial second-degree burns.
Costa de Oliveira Souza [46]	Experimental, rats	Cellulose membranes incorporated with 10% tamarind xyloglucan plus gellan gum 1:1 and 10% lysozyme, or with 10% gellan gum and 10% lysozyme were seeded with allogenic ADSCs and used in a rat burn model. The study group consisted of 40 rats.	No impairment of stem cell activity was observed. The cellulose membrane had a potential antimicrobial activity. Stem cells accelerated epithelialization.	Cellulose membrane is non-toxic for stem cells, and it enables cell proliferation and maturation, as well as migration.
Barrera [47]	Experimental, mice	Allogenic ADSCs were isolated, cultured, enriched with CD26+/CD55+ and seeded in a hydrogel dressing. A contact burn model was established.	Hydrogel seeded with ADSCs healed the wound faster than ADSCs injected. They improved wound vascularization and increased levels of mRNA for *Vegfa* and protein levels for MCP-1, SDF-1, and VEGF. Additionally, the quality of the scar was better with the lower fractional dimension of collagen architecture.	SC-seeded hydrogel significantly improves healing in murine burns.
Dong [48]	Experiment, mice	Hydrogel system comprised of a hyperbranched poly (ethylene glycol) diacrylate (HB-PEGDA) polymer, a commercially available thiol-functionalized hyaluronic acid (HA-SH) and a short RGD peptide enriched in xenogenic ADSC was used to treat second-degree burns in mice.	On day 3, a significant improvement in healing in the treated group was observed. The examination of the specimens showed a higher number of vessels, ratio of collagen type III to I, and reduction of active myofibroblasts.	A novel combined dressing enhanced neovascularization, promoted wound closure and reduced scar formation.
Roshangar [49]	Experimental, rats	Allogenic ADSCs were incorporated in 3D bioprinter-derived gel scaffold and used in scald burn model.	The scaffold was not toxic and did not interfere with ADSC capacities and viability.	3D bioprinter-derived gel scaffold enhanced burn wound treatment.
Razei Yazdi [50]	Experimental, rats	Xenogeneic ADSCs were injected intradermally in 4 areas.	ADSCs reduced inflammatory cells in the wound. They promoted VEGF gene expression and secretion of TGF-β. ADSC also promoted proliferation of dermal fibroblasts.	ADSCs improve burn wound healing by affecting fibroblasts, keratinocytes, and inflammatory cells, as well as increasing the expression of the TGF-β and VEGF genes.
Franck [51]	Experimental, rats	23 rats were used, and one was a donor for ADSCs. A burn model was conducted. ADSCs were injected into the burn wound just after wound cooling. An amount of 3.2 × 10^6^ was used.	ADSCs reduced the burn wound area after 14 days. There was no difference in inflammatory infiltration between the study and control groups. The number of lymphatic vessels was reduced. The concentration of collagen type III was elevated.	ADSCs improve wound healing and reduce scar formation.
Azam [52]	Experimental, rats	ADSCs were isolated from 22 rats, then the isolated cells were incubated in a medium with curcumin for 24 h. An acid burn model was used, 24 h after the injury an excision was made, and the rats were divided into 3 groups. ADSCs were injected intradermally around the wound.	Curcumin improved the healing capacities of ADSCs, increased the capacity of migration, proliferation and paracrine potential, and suppressed secretion of pro-inflammatory cytokines in comparison with innate ADSCs.	Curcumin-preconditioned ADSCs may show potential in the treatment of acid burns.
Babakhani [53]	Experimental, rats	Hair-follicle stem cells were derived from 10 rats. A group of 45 rats was divided into three groups: treatment, control, and sham. A burn model was conducted. The stem cells were injected around the wound bed.	Stem cells improved healing and epidermal thickness. They also stimulated neovascularization and increased the expression of CD31.	Stem cells injected around a deep partial- thickness burn accelerate healing by improvement of epidermal density and neovascularization.
Amini-Nik [54]	Experimental, mice and pigs	Human stem cells were obtained from deeply burned skin. They were used topically in a burn model in 5 mice and 4 pigs.	No tumor formation of stem cells was observed. Stem cells accelerated healing in a mouse and pig model. After 12 days after administration, the stem cells were still present in the wound.	Stem cells obtained from burned skin may be useful in future autologous skin regeneration of deep burns.

**Table 2:** Human studies on stem cells in burn wound healing.

Study	Study Type	Patients & Methods	Outcomes
Kitala [55]	Case report	A 40-year-old patient with deep thermal burns (IIb°/III°-36%TBSA, III°/IV°-1%TBSA). Amnio-derived stem cells were isolated by mechanical homogenization from the placenta and cultured. After bed wound debridement, the wound was covered with stem cells in a saline solution and covered with a dermal matrix substitute.	No adverse events were reported. The wound healed within 12 days. Pain reduction was observed.
Rasulov [56]	Case report	Allogeneic stem cells were collected from two healthy volunteers from the iliac plate. The cells were applied to the surface of the wounds and, after several days, the skin was re-transplanted, this time achieving complete wound closure. A female patient with extensive skin burn (I-II-IIIAB skin burn, total area 40%, area of IIIB degree 30%) was treated.	Rapid healing of the donor site and accelerated healing of deep burns.
Mansilla [57]	Clinical study	Examination of the concentration of stem cells (Flow cytometric analysis, using a large monoclonal antibody panel: CD44, CD45, CD14, DR, CD34, CD19, CD13, CD29, CD105, CD1a, CD90, CD38, CD25. MSC phenotype was considered positive for CD44, CD13, CD29, CD90, and CD105, and negative for the other monoclonals) in the peripheral blood of burned (3 days after injury) and healthy volunteers.	Positive correlation between the number of cells and the extent of the burn. Younger patients had a higher number of stem cells than older patients.
Lattaillade [58]	Case report	A case of severe buttock radiation burns (2000 Gy at the center of the skin surface lesion) in a 27-year-old Chilean. After primary and secondary excisions, bone-marrow-derived mesenchymal stem cells were applied.	Successful treatment of a radiation burn using autogenous myeloid stem cells and a collagen matrix
Jeschke [59]	Case report	A case of a patient with full-thickness burns covering 70% of the body surface, in whom allogeneic myeloid stem cells were used. After debridement of the burn wound, stem cells and fibrin glue were applied to the surface of the burn wound and covered with allografts. About half of the grafts were healed. In the next procedure, the edges of the wound were injected with a commercial suspension of allogeneic myeloid stem cells and the allografts were broken down, with about 90% of the wounds closed as a result.	Stem cells accelerate wound healing.

## Data Availability

Not applicable.
